# Genetic Interactions between Chromosomes 11 and 18 Contribute to Airway Hyperresponsiveness in Mice

**DOI:** 10.1371/journal.pone.0029579

**Published:** 2012-01-10

**Authors:** Caroline M. Ferreira, James L. Chen, Jianrong Li, Kazuhiro Shimomura, Xinan Yang, Yves A. Lussier, Lawrence H. Pinto, Julian Solway

**Affiliations:** 1 Department of Medicine, University of Chicago, Chicago, Illinois, United States of America; 2 Department of Neurobiology and Physiology, Northwestern University, Evanston, Illinois, United States of America; The University of Texas Health Science Center at San Antonio, United States of America

## Abstract

We used two-dimensional quantitative trait locus analysis to identify interacting genetic loci that contribute to the native airway constrictor hyperresponsiveness to methacholine that characterizes A/J mice, relative to C57BL/6J mice. We quantified airway responsiveness to intravenous methacholine boluses in eighty-eight (C57BL/6J X A/J) F_2_ and twenty-seven (A/J X C57BL/6J) F_2_ mice as well as ten A/J mice and six C57BL/6J mice; all studies were performed in male mice. Mice were genotyped at 384 SNP markers, and from these data two-QTL analyses disclosed one pair of interacting loci on chromosomes 11 and 18; the homozygous A/J genotype at each locus constituted the genetic interaction linked to the hyperresponsive A/J phenotype. Bioinformatic network analysis of potential interactions among proteins encoded by genes in the linked regions disclosed two high priority subnetworks - *Myl7*, *Rock1*, *Limk2*; and *Npc1*, *Npc1l1*. Evidence in the literature supports the possibility that either or both networks could contribute to the regulation of airway constrictor responsiveness. Together, these results should stimulate evaluation of the genetic contribution of these networks in the regulation of airway responsiveness in humans.

## Introduction

Airway constrictor hyperresponsiveness (AHR), an exaggerated airway constrictor response to a variety of stimuli, is a defining characteristic of asthma [1], [2]. AHR may represent an underlying genetic predisposition to asthma [3], [4], [5] and can occur independent of atopy [6], [7]. Many studies have focused on the genetic mechanisms that control airway inflammation [see [8] for review], but relatively little attention has been paid to the genetic mechanisms that lead to AHR in the absence of airway inflammation, so-called “native” AHR. The mouse provides an excellent model to investigate the genetic basis for disease predisposition. Because inbred laboratory mice are isogenic and it is possible to control environmental conditions and breeding, studies using mice are subject to less variation from individual-to-individual than occurs in human studies [9]. Moreover, the mouse and human genomes share considerable homology [10], [11]. Thus, valuable insight into human disease can be gained by understanding genetic mechanisms that underlie parallel processes in mice.

Native airway constrictor responsiveness (AR, i.e., in the absence of inflammation) varies widely among inbred mouse strains and is well established as a heritable trait [12], [13]. Among inbred mice, the A/J strain is one of the most responsive and C57BL/6J is one of the least [12], [13], [14], [15]. Quantitative trait locus (QTL) analysis of interbred A/J and C57BL/6J mice identified loci on chromosomes 2, 15, and 17 that individually influence the degree of native AR [14]. Similar analysis of A/J and C3H/HeJ (also hyporesponsive) mice identified additional genes on chromosomes 6 and 7 that also contribute independent influences on AR [15], [16]. Besides these loci that exhibit independent major effects on AR, Beier's group analyzed A/J – C57BL/6J consomic mice, and found that *interacting* loci on chromosomes 2 and 6 also influence AR [17]. Interestingly, the locus on chromosome 6 that contributes independently to AR partially overlaps with the chromosome 6 locus that interacts with chromosome 2 to influence AR.

Here, we have used a different approach to identify a new pair of interacting loci on chromosome 11 and 18 that together, but not individually, influence AR. We performed two-QTL genome scans in second filial generation (F2) mice descended from A/J – C57BL/6J intercrosses, and employed biological network analysis to identify genes within these loci that might interact to determine AR.

## Materials and Methods

### Animals

Male A/J and C57BL/6J mice 7–8 wks of age were purchased from The Jackson Laboratory, Bar Harbor, ME and were bred within our colonies: (C57BL/6J X A/J) F_1_ hybrid, (A/J X C57BL/6J) F_1_ hybrid, (C57BL/6J X A/JF_1_) F_2_ intercross and (A/J X C57BL/6JF_1_) F_2_ intercross. They had free access to food and water and were housed in a temperature-controlled room (22–23°C). The light/dark cycle of the room in which the mice were housed was automatically controlled (10∶14 hrs light∶dark). Mice were weaned at 3 wks age and separated by sex in standard cages. The mice ranged from 22–28 g and 6–8 wk of age at the time of AR phenotyping. All procedures were performed in accordance with NIH and USDA guidelines, and were approved by the Northwestern University Institutional Animal Care and Use Committee (protocol number 2007-0407). The animal facility has been accredited by the Association for Assessment and Accreditation of Laboratory Animal Care-International (AAALAC) and is staffed by three full-time veterinarians. Northwestern University has received assurance from the NIH Office of Protection from Research Risks (PIN-057-2009) and has an Animal Welfare Assurance on file with the Office of Laboratory Animal Welfare (assurance number A3283-01). Reviews of Animal Study Protocols are conducted in accordance with United States Public Health Service (USPHS) regulations and applicable federal and local laws. The composition of the Institutional Animal Care and Use Committee meets the requirements of the USPHS policy and the Animal Welfare Act Regulations.

### Phenotype analysis

All animals were anesthetized with ketamine hydrochloride (100 mg/kg ip) and xylazine (20 mg/kg), and were paralyzed with pancuroniun bromide [18]. A stable depth of anesthesia was maintained by supplemental administration of 30% of the initial anesthesic dose at 25 min intervals. After tracheostomy, the trachea was cannulated with a blunt 18-gauge metal tube, and the mouse was ventilated with a computer controlled small-animal ventilator (flexiVent: Scireq, Montreal, Quebec, Canada) using tidal volume of 10 ml/kg and respiratory frequency of 150 breaths/min. Positive end-expiratory pressure (PEEP) of 2 cm H_2_O was applied throughout. An external jugular vein was isolated for intravenous infusion of methacholine (MCh). At the outset, 6 µg MCh was given iv to ensure that the animal was indeed responsive to MCh and that airway resistance returned to the baseline value after its MCh-induced rise, indicators that the mouse was in stable physiological condition. To obtain a dose-response curve, a bolus of MCh was then injected starting at dose of 1 µg (200 µg/ml solution in PBS; iv boluses of 10–70 µl) and the dose was increased until the airway Newtonian resistance reached a value of 1.0–1.2 cm H_2_O/ml/sec. Thereafter, the maximum dose was given repeatedly (up to 7 times), alternating ventilation pattern between 150 breaths/min, 10 ml/kg and 300 breaths/min, 5 ml/kg with each repetition. We alternated ventilation pattern to test a hypothesis unrelated to that of the current study – that the influence of breathing pattern on airway responsiveness has a genetic basis. However, data gathered during higher frequency, lower tidal volume ventilation were inadequate to address that hypothesis. As such, only Rn measurements obtained during ventilation at 150 breaths/min, 10 ml/kg are reported and analyzed here. Prior to each MCh dose, the expiratory path was obstructed for 15 sec to produce a deep inflation, after which exhalation was immediately allowed. Ventilation was continued for about 2 min between consecutive MCh doses. Airway responsiveness was quantified as the average of 2 to 4 measurements of Newtonian resistance (Rn) at the highest MCh dose given, divided by the quantity (MCh dose/mouse weight); i.e., airway responsiveness = Rn/(dose/mouse weight).

### Genotype analysis

Genomic DNA was isolated from tails and purified using the Gentra Puregene Cell and Tissue kit according to the manufacturer's instructions. Spectrophotometric readings of DNA samples were made to verify purity (ratio of absorbance at 260 nm to that at 280 nm) and to quantify the DNA concentration (A_260_). Genotype analysis was performed by the Mutation Mapping and Development Analysis Project (MMDAP; Brigham and Women's Hospital, Harvard Medical School), which provided a fixed whole genome panel of 384 evaluable mouse SNPs (polymorphic for our cross: A/J X C57BL/6J) genotyped on the Illumina genotyping platform. This panel has an average SNP density of 3 Mb across autosomes and 7 Mb across chromosome X [19].

### QTL analysis

**Figure 1:**
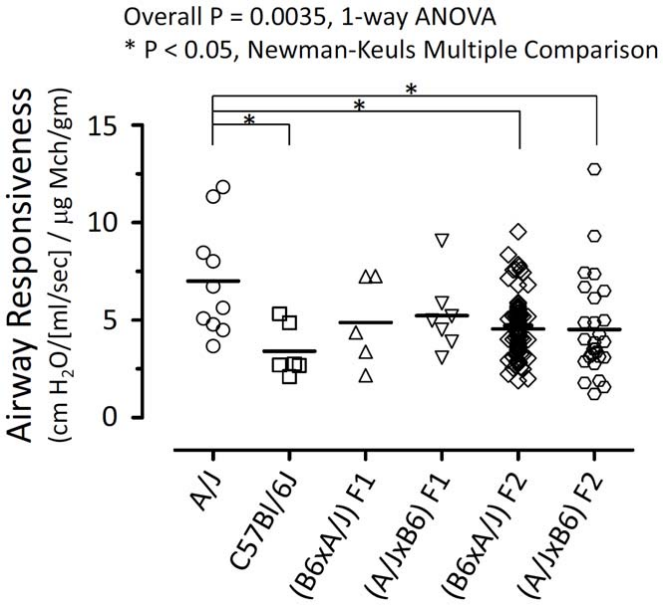
Airway responsiveness of mice from parental strains, F1 generations, or F2 generations as shown. Each individual symbol represents the datum of a single mouse; horizontal lines show mean values. Airway responsiveness is calculated as Newtonian resistance at the highest dose of methacholine, divided by that dose of methacholine normalized to body weight. B6 - C57BL/6J.

To locate main effect and interacting QTLs linked to native airway responsiveness, we performed statistical analysis using R/qtl software [20]. Functions for estimating genetic maps, identifying genotyping errors, and performing single-QTL and two-dimensional, two-QTL genome scans are included in the software (http://www.rqtl.org). Data used in these genetic analyses were obtained from the 115 F2 mice, plus both parental strains. To avoid overrepresentation of parental strains in the genetic analysis, we included one mouse of each parental strain in our analysis, using the average value of airway responsiveness for that strain as calculated in Figure 1. Thus, the genetic analysis was performed using data from 117 mice – 115 F2 mice plus one “average” A/J mouse and one “average” C57BL/6J mouse.

### Mapping of chromosomal regions to gene lists

**Figure 2:**
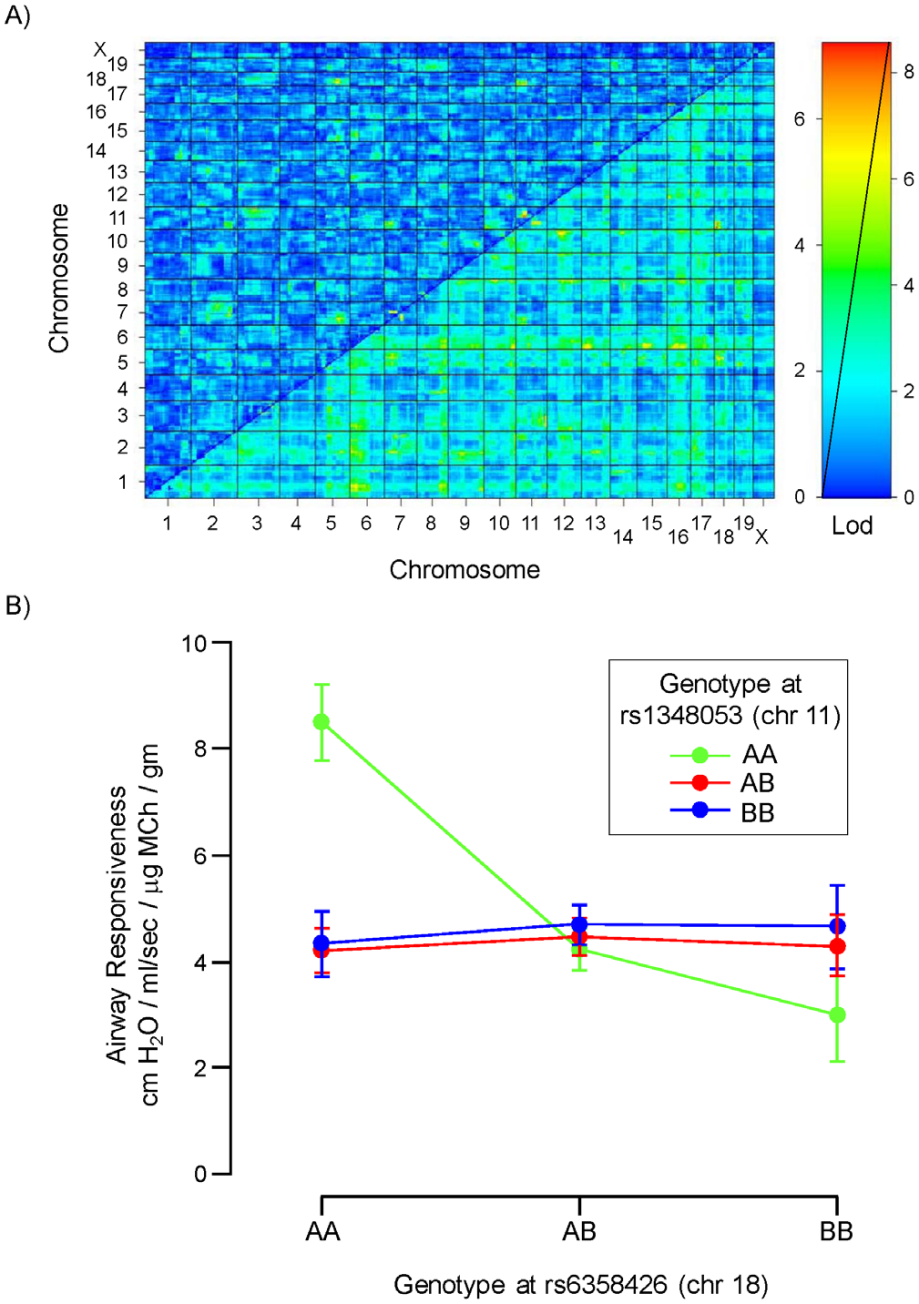
Genetic factors that influence airway responsiveness. A) Heat map showing lod scores derived from two-QTL (upper left half) and one-QTL (lower right half) analyses for linkage to airway responsiveness in 115 F2 mice and one mouse of each parental strain (using mean values of airway responsiveness for A/J and C57Bl/6J mice, as calculated in Figure 1). B) Genotypes at markers rs13480853 on chromosome 11 and rs6358426 on chromosome 18 interact significantly to determine native airway responsiveness. Mice with homozygous A/J genotype at both loci exhibit significantly greater AR (about double) than mice with any other sets of genotypes. Mean ± SEM shown. AA - homozygous A/J genotype; AB - heterozygous A/J+C57BL/6J genotype; BB - homozygous C57BL/6J genotype.

As shown in Figure 2, our two-QTL analysis revealed loci on chromosomes 11 (peak linkage at rs13480853) and 18 (peak linkage at rs6358426) that interact to determine AR; furthermore, the interaction that resulted in the high AR phenotype required a homozygous A/J polymorphism at both loci. To identify the extent of linkage disequilibrium, we analyzed the haplotypes of the 6 animals that exhibited high AR and homozygous A/J genotype at the peak linkage markers; we considered the linked locus to extend throughout the span in which no cross-over had occurred in any high AR mouse, plus 1 MB beyond the point at which cross-over had occurred, to account for potential gene expression regulatory elements that can occur relatively distant to the coding exons [21]. Candidate gene lists in each of the interacting linked spans were constructed from the Mouse Genome Informatics (MGI) database (www.informatics.jax.org) and among these, potentially “directly” interacting genes or gene products were identified using protein interaction databases.

### Protein Interaction Network Analysis of Candidate Genes

To analyze the interactions of the candidate genes within linked regions, we first generated a protein-protein interaction network (PPIN) derived from the Search Tool for the Retrieval of Interacting Genes version 8.1 (STRING; http://string-db.org) [22]. We extracted all mouse interactions and we retained those interactions with a combined score of >800 (highly reliable score) that also comprised either gene fusion, experimental or database evidence. Therefore, literature-based protein interactions were filtered out, resulting in a PPIN of 77,886 interactions containing 5,830 distinct proteins based on the UniProt standard (www.uniprot.org).

Two datasets containing target genes from the areas of interest on chromosome 18 (1 Mb telomeric of the interval from rs13483183 to rs6358426) and chromosome 11 (1 Mb telomeric of the interval from rs3659787 to rs13480853) were then compared using the Single Protein Analysis of Network (SPAN) algorithm which has been described previously [23]. In brief, SPAN uses a link-randomization method that conserves the number of “connections” of each specific protein (node-degree) and estimates the probability of interaction of each protein encoded in the linked region of chromosome 11 with every protein encoded in the linked region of chromosome 18, and vice versa. Since each protein conserves the same number of interactors in each synthetic network, a probability of occurrence of interaction can be derived for the observed number of interactors for each protein (node) [23]. We extended the SPAN metric to also estimate a probability of occurrence of a single interaction (i.e., an “edge”) between two specific proteins, one from each linked chromosomal region. These probabilities for node and edge interactions are then further controlled for multiplicity using false discovery rate corrections. Cytoscape software was then used to display these interactions [24].

## Results

### Distribution of native AR among parental strains and their F_1_ and F_2_ progeny

Airway responsiveness was measured in ten A/J, six C57BL/6J, five (C57BL/6J X A/J) F_1_, six (A/J X C57BL/6J) F_1_, eighty-eight (C57BL/6J X A/J) F_2_, and twenty-seven (A/J X C57BL/6J) F_2_ mice. Because age, gender, and airway infection can alter AR, only 7–8 wk old male mice raised under barrier conditions were studied. Consistent with previous findings [16], we found that A/J mice have a statistically significantly increased level of AR compared with C57BL/6J mice or with mice in either F2 group (Figure 1) (P = 0.0035, ANOVA among all 6 groups; P<0.05, Newman-Keuls Multiple Comparisons test, for A/J vs C57BL/6J and A/J vs either F2 group). However, in contrast to previous reports [16], [17], we did not find the A/J phenotype to be dominant in F1 mice (Figure 1); rather, F1 mice exhibited AR that was intermediate between that of each parental strain. Furthermore, we found elevated AR not to be sex linked, since (A/J X C57BL/6J) F_1_ and (C57BL/6J X A/J) F_1_ mice had indistinguishable phenotypes. AR values for F_2_ mice were approximately normally distributed (Figure 1), consistent with previous findings that AR is under the control of multiple genes [15], [16], [17].

### QTL analysis

To carry out a genome-wide scan for genetic factors that influence AR, we used 384 SNP genotypes of the 115 F2 mice studied, as well as those of the parental A/J and C57BL/6J strains (mean values of AR were used for parental strains). The single QTL genome scan (Table 1) showed that maximum lod scores occurred for the following markers: rs13478612 (chr 6, lod = 2.1), rs3693295 (chr 8 lod = 2.4), rs13480777 (chr 10, lod = 2.286) and rs13459150 (chr 17, lod = 2.0). Some of these loci are located closed to regions that were previously implicated in regulating AR. For example, rs13478612 is close to the Bhr5 locus and rs13459150 is close to the Bhr3 locus [16]. It is noteworthy that none of these loci exhibited a lod score sufficient to consider significant linkage established at any individual locus. In contrast, we did find one significant interaction between a locus on chr 11 (rs13480853) and another locus on 18 (rs6358426) with lod interaction = 6.8 (significant interaction requires lod>6.3, as calculated by permutation test in R/qtl) (Table 1). In this interaction, the high AR phenotype was associated with the homozygous A/J genotype at both loci (n = 6 animals) (Figure 2). Two-way ANOVA of the data shown in Figure demonstrated significant interaction between the genotypes at these chr 11 and chr 18 markers (P<0.0001), and one-way ANOVA (P<0.0001 among all genotype combinations) followed by Newman-Keuls Multiple Comparison test revealed that mice homozygous for the A/J genotype at both loci have significantly greater airway responsiveness than mice of any other genotype combination (P<0.05, 11-AA/18-AA vs all other genotypes at these loci).

**Table 1:** Lod scores for linkage to AR.

SNP	Chr	Lod score
rs13478612	6	2.1
rs3693295	8	2.4
rs13480777	10	2.2
rs13459150	17	2
rs3674810+rs3661724	3+11	5.2
rs3725746+rs13479208	5+7	5
rs13478240+rs13481117	5+11	5.22
rs3725746+rs13483280	5+18	5
rs4139111+rs3701609	11+11	5.26
rs13480853+rs6358426	11+18	6.8

### Protein network analysis

To identify potentially interacting pairs of genes within the linked pairs of loci that influence AR, we first needed to determine the extent of the non-recombinant locus. To do this, we analyzed the haplotype of the 6 animals (5 F2 mice and A/J parental strain) that demonstrated the high AR phenotype and were homozygous A/J genotype at both interacting loci. This analysis showed that the non-recombinant region extended from marker rs3659787 to rs13480853 on chromosome 11 and from rs13483183 to rs6358426 on chromosome 18 (Table 2). Because cis-acting control elements are often as far as 1 Mb [21] outside of the transcribed exons, we analyzed the interactions between proteins encoded by genes in each non-recombinant region plus 1 Mb on either end (listed in Table S1) using the bioinformatics methods described above.

**Table 2:** Genotypes of mice with AA genotypes at rs13480853 and rs6358426.

SNP at chr 11	Mouse 1	Mouse 2	Mouse 3	Mouse 4	Mouse 5	Mouse 6
rs13480836	AA	AA	AB	AA	AA	AA
rs3659787	AA	AA	AA	AA	AA	AA
rs13480853	AA	AA	AA	AA	AA	AA
rs6398304	AA	AA	AA	AA	BB	AA

SNP at chr 18	Mouse 1	Mouse 2	Mouse 3	Mouse 4	Mouse 5	Mouse 6
rs13483183	AA	AA	AA	AA	AA	AA
rs3660245	AA	AA	AA	AA	AA	AA
rs6358426	AA	AA	AA	AA	AA	AA
rs13483251	AA	AA	AB	AA	AA	AA

Underlined markers (rs13480853 and rs6358426) represent the SNPs that provide significant evidence for interaction to determine airway responsiveness.

**Figure 3:**
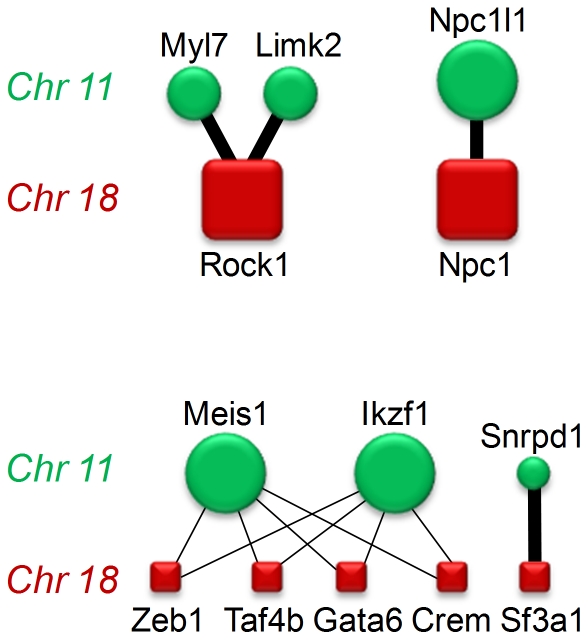
Network prioritization of potential interactions between proteins encoded by genes located within the linked regions on chromosomes 11 and 18. Proteins and their interactions were prioritized using extended SPAN modeling as described in Methods. Among all potential interactions, only two networks (*Limk2*, *Rock1*, *Myl7*; and *Npc1*, *Npc1l1*) included prioritization of both proteins (nodes) and their interactions (edges) at a false discovery rate of less than 0.0001. Every protein-protein interaction in which at least one node or one edge was prioritized is displayed. Prioritization is denoted as follows: Large genes and thick interaction lines - false discovery rate (FDR)<0.0001; medium size genes - FDR<0.5; small genes and thin interaction lines - FDR>0.5.

From these candidate genes, UniProt listed proteins (excluding ESTs, predicted genes, microRNAs) were extracted. One hundred fifty-eight distinct genes were encoded on chromosome 11 and 83 distinct genes on chromosome 18; we retained these UniProt proteins for further analysis. Of these proteins, eight proteins encoded on chromosome 11 and eight encoded on chromosome 18 were connected by fourteen unique protein interactions in the STRING network; these interactions then served as seed data for our subsequent SPAN prioritization. Using the Single Protein Analysis of Network algorithm [23] over the protein-protein interaction network, two networks emerged as possessing a false discovery rate of less than 0.0001 at both the interaction (edge) and protein (node) levels; these were: 1) *Limk2*, *Rock1*, and *Myl7*; and 2) *Npc1* and *Npc1l1* (Figure 3). Importantly, these genes are polymorphic between A/J and C57BL/6J strains, as delineated in the Jackson Laboratory Mouse Phenome Database and summarized in Table S2.

## Discussion

Most previous studies addressing the genetic basis of native airway responsiveness (AR) in mice have identified loci that exert independent (non-interacting) influences on AR; these loci occur on chromosomes 1, 2, 3, 5, 6, 7, 8, 10, 12, 13, 15, and 17 [25], [26]. In addition, one prior study [17] addressed interactions among different chromosomal loci in determining AR in A/J vs C57BL/6J mice, and using consomic mice demonstrated that regions of chromosomes 2 and 6 interact to contribute to AR differences between these strains. Our study extends current understanding of the genetic basis underlying native AR in mice by demonstrating a new pair of interacting loci on chromosomes 11 and 18 that strongly influence AR in F2 progeny of A/J and C57BL/6J mice (Figure 2). Interestingly, neither of these novel loci contributes significantly as an independent determinant of AR.

To determine which genes within the linked interacting loci are likely to account for their biological interaction, we used a bioinformatics approach in which all known proteins encoded by genes within the linked locus on chromosome 11 were analyzed for confirmed direct interactions with all known proteins encoded by genes within the linked locus on chromosome 18, and vice versa, at both the individual protein (node) and interacting proteins (edge) levels. This analysis disclosed only 4 networks of interacting proteins (Figure 3), among which only two networks contained both individual proteins and protein-protein interactions prioritized with false discovery rates of less than 0.0001 - *Limk2*, *Rock1*, *Myl7*; and *Npc1*, *Npc1l1*. Each of these networks could plausibly be involved in regulating AR.

In the *Limk2*, *Rock1*, *Myl7* network, each of the chromosome 11 gene products (*Myl7* and *Limk2*) was prioritized with false discovery rate less than 50%. As such, it is likely that only one of the two proteins is the important interactor with *Rock1* in determining the native airway hyperresponsiveness seen in A/J mice. Since *Myl7* encodes the cardiac atrial isoform of myosin regulatory light chain, it seems more likely that *Limk2* interacts with *Rock1* to determine the constrictor responsiveness of pulmonary airways to methacholine. *Rock1* encodes rho kinase, an enzyme expressed in airway smooth muscle that promotes actin filament polymerization by phosphorylating and activating Lim kinase (the enzyme encoded by *Limk2*), which in turn phosphorylates and thereby inactivates cofilin, whose function promotes actin filament depolymerization. Actin polymerization plays critical roles [27], [28], [29] in airway smooth muscle contraction. As such, we think it plausible that A/J mice express genetic variants or abundances of Rock1 and Limk2 proteins that uniquely interact to accentuate actin polymerization during contraction. Thus, having only the A/J variant of each gene might result in enhanced contractile function or in reduced force fluctuation-induced relengthening of contracting airway smooth muscle [30], [31]. These functional consequences might result in the native airway constrictor hyperresponsiveness so characteristic of A/J mice. It is also conceivable that the A/J variant of *Limk2* potentiates other functions of A/J rho kinase. Importantly, rho kinase also promotes smooth muscle contraction [32] by phosphorylating the myosin targeting subunit of myosin light chain phosphatase thereby promoting the phosphorylated state of myosin regulatory light chain, and perhaps also by promoting focal adhesion assembly.

In the *Npc1*, *Npc1l1* network, each gene individually and the genes' interaction were prioritized with false discovery rate less than 0.0001. *Npc1* encodes a protein located in the membranes of lysosomes and endosomes, and is thought to regulate intracellular trafficking of cholesterol. Mutations of *Npc1* are responsible for most cases of Niemann-Pick disease type C, which is associated with abnormal accumulation of cholesterol and other lipids within cells. Niemann-Pick disease can involve the lung with foam cell infiltration [33], [34], [35], but airway hyperresponsiveness has not been reported. *Npc1l1* is a separate gene expressed in gut epithelium [36], [37], [38], [39], [40], [41] whose protein product regulates cholesterol absorption, thereby influencing whole body cholesterol homeostasis, and is the target of ezetimibe. A growing body of literature implicates cholesterol or modulators of metabolism in the regulation native airway responsiveness and/or allergic airway inflammation [42], [43], [44], [45], [46], [47]. Depletion of cholesterol from membrane caveoli disrupts contractile activation of airway smooth muscle during muscarinic, serotonin, or KCl-stimulation [48], [49], and further inhibits rho kinase activation [48]. As such, strain-related variations in two genes, *Npc1* and *Npc1l1*, which each influence cholesterol homeostasis could plausibly interact to regulate AR in A/J vs C57BL/6J mice.

There are potential limitations to our study. We tested only male mice, and so might have missed the opportunity to detect other interacting gene pairs that might be important in females. There were other pairs of interacting loci that were suggested by our two-QTL analysis as potential regulators of AR, but these did not reach statistical significance (Table 1). Perhaps one or more of these pairs would have emerged as statistically significant had we phenotyped and genotyped a much larger number of F2 mice. Lastly, we used a novel measure of airway responsiveness - Newtonian resistance (Rn) at the highest MCh dose given, divided by Mch dose and divided by mouse weight, when methacholine dose was titrated to achieve Rn of approximately 1 cm H_2_O/ml/sec. We chose this approach because it allowed use to evaluate airway responsiveness at approximately the same level of bronchoconstriction in each mouse.

In summary, two-QTL analysis disclosed an interacting pair of novel loci that contribute to the native airway hyperresponsiveness in A/J mice. Further bioinformatics analysis suggests that the presence of homozygous A/J alleles of *Rock1* and *Limk2*, or of *Npc1* and *Npc1l1*, or of both pairs, may account for the observed genetic interaction, and evidence from the literature supports the plausibility of these possibilities. Definitive testing of these possibilities could be done by evaluating the airway responsiveness of sets of genetically engineered mice that harbor the A/J or C57BL/6J alleles of either or both genes of each network pair, set in either the A/J or C57BL/6J background. Our present results should stimulate evaluation of the genetic contribution of these networks in the regulation of airway responsiveness in humans.

## Supporting Information

Table S1 **Genes within linke regions on chromosomes 11 and 18.**
(DOC)

Table S2 **A/J vs C57BL/6J polymorphisms from Jackson Laboratory Mouse Phenome Database (** http://phenome.jax.org/SNP **).**
(DOC)
