# Supplementary material for: Genetic Interactions between Chromosomes 11 and 18 Contribute to Airway Hyperresponsiveness in Mice
Source: PLoS One. 2012 Jan 10;7(1):e29579. doi: 10.1371/journal.pone.0029579 (PMC3254621; doi:10.1371/journal.pone.0029579)
Supplement: Table S1 — Genes within linke regions on chromosomes 11 and 18. (DOC) [file pone.0029579.s001.doc]

| **Chromosome 11** | |
| --- | --- |
| *Abca13* | ATP-binding cassette, sub-family A (ABC1), member 13 |
| *Actr2* | ARP2 actin-related protein 2 homolog (yeast) |
| *Adcy1* | adenylate cyclase 1 |
| *Aebp1* | AE binding protein 1 |
| *Aftph* | aftiphilin |
| *Ahsa2* | AHA1, activator of heat shock protein ATPase homolog 2 (yeast) |
| *Akt2-ps* | thymoma viral proto-oncogene 2, pseudogene |
| *Ankrd36* | ankyrin repeat domain 36 |
| *Ap1b1* | adaptor protein complex AP-1, beta 1 subunit |
| *Ascc2* | activating signal cointegrator 1 complex subunit 2 |
| *B3gnt2* | UDP-GlcNAc:betaGal beta-1,3-N-acetylglucosaminyltransferase 2 |
| *Bcl11a* | B-cell CLL/lymphoma 11A (zinc finger protein) |
| *Bglq8* | body growth late QTL 8 |
| *Bits3* | bitterness sensitivity 3 |
| *Bmch5* | bone mechanical trait 5 |
| *Brnvl1* | brain volume 1 |
| *C1d* | C1D nuclear receptor co-repressor |
| *Cabp7* | calcium binding protein 7 |
| *Camk2b* | calcium/calmodulin-dependent protein kinase II, beta |
| *Ccdc117* | coiled-coil domain containing 117 |
| *Ccdc157* | coiled-coil domain containing 157 |
| *Ccm2* | cerebral cavernous malformation 2 homolog (human) |
| *Cct4* | chaperonin containing Tcp1, subunit 4 (delta) |
| *Cep68* | centrosomal protein 68 |
| *Cnrip1* | cannabinoid receptor interacting protein 1 |
| *Cobl* | cordon-bleu |
| *Commd1* | COMM domain containing 1 |
| *Dbnl* | drebrin-like |
| *Ddc* | dopa decarboxylase |
| *Ddx56* | DEAD (Asp-Glu-Ala-Asp) box polypeptide 56 |
| *Drg1* | developmentally regulated GTP binding protein 1 |
| *Dusp18* | dual specificity phosphatase 18 |
| *E230015J15Rik* | RIKEN cDNA E230015J15 gene |
| *Eae6a* | experimental allergic encephalomyelitis susceptibility 6a |
| *Egfr* | epidermal growth factor receptor |
| *Ehbp1* | EH domain binding protein 1 |
| *Eif4enif1* | eukaryotic translation initiation factor 4E nuclear import factor 1 |
| *Emid1* | EMI domain containing 1 |
| *Etaa1* | Ewing's tumor-associated antigen 1 |
| *Ewsr1* | Ewing sarcoma breakpoint region 1 |
| *Fam161a* | family with sequence similarity 161, member A |
| *Fau-ps2* | Finkel-Biskis-Reilly murine sarcoma virus (FBR-MuSV) ubiquitously expressed (fox derived), pseudogene 2 |
| *Fbxo48* | F-box protein 48 |
| *Fignl1* | fidgetin-like 1 |
| *Gal3st1* | galactose-3-O-sulfotransferase 1 |
| *Gas2l1* | growth arrest-specific 2 like 1 |
| *Gatsl3* | GATS protein-like 3 |
| *Gck* | glucokinase |
| *Glns-ps1* | glutamine synthetase pseudogene 1 |
| *Gluchos2* | glucose homeostasis QTL 2 |
| *Grb10* | growth factor receptor bound protein 10 |
| *H2afv* | H2A histone family, member V |
| *Hemq4* | hematocrit QTL 4 |
| *Hipp5* | hippocampal weight 5 |
| *Hormad2* | HORMA domain containing 2 |
| *Hus1* | Hus1 homolog (S. pombe) |
| *Igf1sl4* | IGF-1 serum levels 4 |
| *Igfbp1* | insulin-like growth factor binding protein 1 |
| *Igfbp3* | insulin-like growth factor binding protein 3 |
| *Ikzf1* | IKAROS family zinc finger 1 |
| *Inpp5j* | inositol polyphosphate 5-phosphatase J |
| *Kremen1* | kringle containing transmembrane protein 1 |
| *Lif* | leukemia inhibitory factor |
| *Limk2* | LIM motif-containing protein kinase 2 |
| *Lith23* | lithogenic gene 23 |
| *Lmr6* | leishmaniasis resistance 6 |
| *Manln10* | mandible length 10 |
| *Mcvq2* | mean corpuscular volume QTL 2 |
| *Mdh1* | malate dehydrogenase 1, NAD (soluble) |
| *Meis1* | Meis homeobox 1 |
| *Mndl1* | mandible length 1 |
| *Morc2a* | microrchidia 2A |
| *Mrps24* | mitochondrial ribosomal protein S24 |
| *Mtmr3* | myotubularin related protein 3 |
| *Myl7* | myosin, light polypeptide 7, regulatory |
| *Myo1g* | myosin IG |
| *Nacad* | NAC alpha domain containing |
| *Nefh* | neurofilament, heavy polypeptide |
| *Nf2* | neurofibromatosis 2 |
| *Nidd4n* | non-insulin-dependent diabetes mellitus 4 in NSY |
| *Nipsnap1* | 4-nitrophenylphosphatase domain and non-neuronal SNAP25-like protein homolog 1 (C. elegans) |
| *Npc1l1* | *NPC1*-like 1 |
| *Nudcd3* | NudC domain containing 3 |
| *Ogdh* | oxoglutarate dehydrogenase (lipoamide) |
| *Orgwq8* | organ weight QTL 8 |
| *Osbp2* | oxysterol binding protein 2 |
| *Osm* | oncostatin M |
| *Otx1* | orthodenticle homolog 1 (Drosophila) |
| *Papolg* | poly(A) polymerase gamma |
| *Patz1* | POZ (BTB) and AT hook containing zinc finger 1 |
| *Peli1* | pellino 1 |
| *Pes1* | pescadillo homolog 1, containing BRCT domain (zebrafish) |
| *Pex13* | peroxisomal biogenesis factor 13 |
| *Pgam1-ps1* | phosphoglycerate mutase 1, pseudogene 1 |
| *Pgam2* | phosphoglycerate mutase 2 |
| *Pik3ip1* | phosphoinositide-3-kinase interacting protein 1 |
| *Pisd-ps1* | phosphatidylserine decarboxylase, pseudogene 1 |
| *Pkd1l1* | polycystic kidney disease 1 like 1 |
| *Pla2g3* | phospholipase A2, group III |
| *Plek* | pleckstrin |
| *Pno1* | partner of NOB1 homolog (S. cerevisiae) |
| *Pold2* | polymerase (DNA directed), delta 2, regulatory subunit |
| *Polm* | polymerase (DNA directed), mu |
| *Pom121l12* | POM121 membrane glycoprotein-like 12 |
| *Ppia* | peptidylprolyl isomerase A |
| *Ppp3r1* | protein phosphatase 3, regulatory subunit B, alpha isoform (calcineurin B, type I) |
| *Purb* | purine rich element binding protein B |
| *Pus10* | pseudouridylate synthase 10 |
| *Rab1* | RAB1, member RAS oncogene family |
| *Ramp3* | receptor (calcitonin) activity modifying protein 3 |
| *Rasl10a* | RAS-like, family 10, member A |
| *Rel* | reticuloendotheliosis oncogene |
| *Rhbdd3* | rhomboid domain containing 3 |
| *Rnf185* | ring finger protein 185 |
| *Rnf215* | ring finger protein 215 |
| *Scc6* | colon tumor susceptibility 6 |
| *Sec14l2* | SEC14-like 2 (S. cerevisiae) |
| *Sec14l3* | SEC14-like 3 (S. cerevisiae) |
| *Sec14l4* | SEC14-like 4 (S. cerevisiae) |
| *Sec61g* | SEC61, gamma subunit |
| *Selm* | selenoprotein M |
| *Sertad2* | SERTA domain containing 2 |
| *Sf3a1* | splicing factor 3a, subunit 1 |
| *Sfi1* | Sfi1 homolog, spindle assembly associated (yeast) |
| *Skull15* | skull morphology 15 |
| *Slc1a4* | solute carrier family 1 (glutamate/neutral amino acid transporter), member 4 |
| *Slc35e4* | solute carrier family 35, member E4 |
| *Smtn* | smoothelin |
| *Snora5c* | small nucleolar RNA, H/ACA box 5C |
| *Spred2* | sprouty-related, EVH1 domain containing 2 |
| *Sunc1* | Sad1 and UNC84 domain containing 1 |
| *Tbc1d10a* | TBC1 domain family, member 10a |
| *Tbrg4* | transforming growth factor beta regulated gene 4 |
| *Tcn2* | transcobalamin 2 |
| *Thoc5* | THO complex 5 |
| *Tmed4* | transmembrane emp24 protein transport domain containing 4 |
| *Tmem17* | transmembrane protein 17 |
| *Tns3* | tensin 3 |
| *Tshp9* | tooth shape 9 |
| *Tug1* | taurine upregulated gene 1 |
| *Ugp2* | UDP-glucose pyrophosphorylase 2 |
| *Upp1* | uridine phosphorylase 1 |
| *Usp34* | ubiquitin specific peptidase 34 |
| *Vps54* | vacuolar protein sorting 54 (yeast) |
| *Vstm2a* | V-set and transmembrane domain containing 2A |
| *Vtbt11* | vertebral trabecular bone trait 11 |
| *Vwc2* | von Willebrand factor C domain containing 2 |
| *W6q3* | weight 6 weeks QTL 3 |
| *Wap* | whey acidic protein |
| *Wdr92* | WD repeat domain 92 |
| *Xbp1* | X-box binding protein 1 |
| *Xpo1* | exportin 1, CRM1 homolog (yeast) |
| *Ykt6* | YKT6 homolog (S. Cerevisiae) |
| *Zmat5* | zinc finger, matrin type 5 |
| *Zmiz2* | zinc finger, MIZ-type containing 2 |
| *Znrf3* | zinc and ring finger 3 |
| *Zpbp* | zona pellucida binding protein |
| *Zrsr1* | zinc finger (CCCH type) |

| **Chromosome 18** | |
| --- | --- |
| *Abhd3* | abhydrolase domain containing 3 |
| *AK220484* | cDNA sequence AK220484 |
| *Ankrd29* | ankyrin repeat domain 29 |
| *Aqp4* | aquaporin 4 |
| *Arhgap12* | Rho GTPase activating protein 12 |
| *Armc4* | armadillo repeat containing 4 |
| *Asxl3* | additional sex combs like 3 (Drosophila) |
| *AW742475* | expressed sequence AW742475 |
| *B4galt6* | UDP-Gal:betaGlcNAc beta 1,4-galactosyltransferase, polypeptide 6 |
| *Bambi* | BMP and activin membrane-bound inhibitor, homolog (Xenopus laevis) |
| *Brml7* | bone response to mechanical loading 7 |
| *Cables1* | CDK5 and Abl enzyme substrate 1 |
| *Cabyr* | calcium-binding tyrosine-(Y)-phosphorylation regulated (fibrousheathin 2) |
| *Ccny* | cyclin Y |
| *Cdh2* | cadherin 2 |
| *Cetn1* | centrin 1 |
| *Chst9* | carbohydrate (N-acetylgalactosamine 4-0) sulfotransferase 9 |
| *Colec12* | collectin sub-family member 12 |
| *Crem* | cAMP responsive element modulator |
| *Cul2* | cullin 2 |
| *Dsc1* | desmocollin 1 |
| *Dsc2* | desmocollin 2 |
| *Dsc3* | desmocollin 3 |
| *Dsg1a* | desmoglein 1 alpha |
| *Dsg1b* | desmoglein 1 beta |
| *Dsg1c* | desmoglein 1 gamma |
| *Dsg2* | desmoglein 2 |
| *Dsg3* | desmoglein 3 |
| *Dsg4* | desmoglein 4 |
| *Dtna* | dystrobrevin alpha |
| *Epc1* | enhancer of polycomb homolog 1 (Drosophila) |
| *Esco1* | establishment of cohesion 1 homolog 1 (S. cerevisiae) |
| *Fabp5l2* | fatty acid binding protein 5-like 2 |
| *Fam59a* | family with sequence similarity 59, member A |
| *Fzd8* | frizzled homolog 8 (Drosophila) |
| *Gata6* | GATA binding protein 6 |
| *Gjd4* | gap junction protein, delta 4 |
| *Hrh4* | histamine receptor H4 |
| *Idd21c* | insulin dependent diabetes susceptibility 21c |
| *Impact* | imprinted and ancient |
| *Kctd1* | potassium channel tetramerisation domain containing 1 |
| *Kif5b* | kinesin family member 5B |
| *Klhl14* | kelch-like 14 (Drosophila) |
| *Lama3* | laminin, alpha 3 |
| *Lyzl1* | lysozyme-like 1 |
| *Map3k8* | mitogen-activated protein kinase kinase kinase 8 |
| *Mapre2* | microtubule-associated protein, RP/EB family, member 2 |
| *Mep1b* | meprin 1 beta |
| *Mib1* | mindbomb homolog 1 (Drosophila) |
| *Mir1-2* | microRNA 1-2 |
| *Mir1-2as* | microRNA 1-2, antisense |
| *Mir133a-1* | microRNA 133a-1 |
| *Mir1948* | microRNA 1948 |
| *Mkx* | mohawk homeobox |
| *Mom7* | modifier of Min 7 |
| *Mpp7* | membrane protein, palmitoylated 7 (MAGUK p55 subfamily member 7) |
| *Mtpap* | mitochondrial poly(A) polymerase |
| *Nol4* | nucleolar protein 4 |
| *Npc1* | Niemann Pick type C1 |
| *Osbpl1a* | oxysterol binding protein-like 1A |
| *Otx2m1* | orthodenticle homolog 2 (Drosophila) modifier 1 |
| *Psma8* | proteasome (prosome, macropain) subunit, alpha type, 8 |
| *Rab18* | RAB18, member RAS oncogene family |
| *Rafaril* | Rafar interacting locus |
| *Raml1* | radiation induced acute myeloid leukemia 1 |
| *Rbbp8* | retinoblastoma binding protein 8 |
| *Riok3* | RIO kinase 3 (yeast) |
| *Rnf125* | ring finger protein 125 |
| *Rnf138* | ring finger protein 138 |
| *Rock1* | Rho-associated coiled-coil containing protein kinase 1 |
| *Snrpd1* | small nuclear ribonucleoprotein D1 |
| *Ss18* | synovial sarcoma translocation, Chromosome 18 |
| *Svil* | supervillin |
| *Taf4b* | TAF4B RNA polymerase II, TATA box binding protein (TBP)-associated factor |
| *Thoc1* | THO complex 1 |
| *Tpi-rs10* | triosephosphate isomerase related sequence 10 |
| *Ttc39c* | tetratricopeptide repeat domain 39C |
| *Ttr* | transthyretin |
| *Usp14* | ubiquitin specific peptidase 14 |
| *Wac* | WW domain containing adaptor with coiled-coil |
| *Zeb1* | zinc finger E-box binding homeobox 1 |
| *Zfp438* | zinc finger protein 438 |
| *Zfp521* | zinc finger protein |
