# Supplementary material for: Genetic Interactions between Chromosomes 11 and 18 Contribute to Airway Hyperresponsiveness in Mice
Source: PLoS One. 2012 Jan 10;7(1):e29579. doi: 10.1371/journal.pone.0029579 (PMC3254621; doi:10.1371/journal.pone.0029579)
Supplement: Table S2 — A/J vs C57BL/6J polymorphisms from Jackson Laboratory Mouse Phenome Database ( http://phenome.jax.org/SNP ). (DOC) [file pone.0029579.s002.doc]

|  | | | | | |  |  |
| --- | --- | --- | --- | --- | --- | --- | --- |
| Mbp | NCBI | Ensembl | dbSNP | A/J | C57BL  /6J | dbSNP | Source |
| location | gene | 48 | 128 |  |  | rs |  |
| (Build 37) | annotation | gene | SNP |  |  |  |  |
|  |  | annotation | annotation |  |  |  |  |
|  |  |  |  |  |  |  |  |
| ***Rock1*** |  |  |  |  |  |  |  |
| 18 10.116541 | [*Rock1*   intron10](javascript:;) | ... intron12 | I | C | G | rs30210333 | Perlegen2   NES12690008 |
| 18 10.144887 |  | [*Rock1* intron2](javascript:;) | *Rock1* I | C | T | rs30984994 | Perlegen2   NES12689250 |
|  |  |  | LOC100039978 L |  |  |  |  |
|  |  |  |  |  |  |  |  |
| ***Limk2*** |  |  |  |  |  |  |  |
| 11 3.255342 | [*Limk2*   exon9](javascript:;) | ... exon3 | **Cn**   T262M | A | G | rs26884114 | Perlegen2   NES08504913 |
| 11 3.255387 | [*Limk2*   exon9](javascript:;) | ... exon3 | **Cn**   Q247R | T | C | rs26884113 | (Celera2,Perlegen2) |
| 11 3.267480 | [*Limk2*   intron3](javascript:;) | ... intron1 | I | C | A | rs26884078 | Perlegen2   NES08504850 |
|  |  |  |  |  |  |  |  |
| ***Myl7*** |  |  |  |  |  |  |  |
| 11 5.795300 |  |  | *Myl7* L | A | T | rs29384719 | Celera2   mCV22906378 |
| 11 5.795462 |  |  | *Myl7* L | T | A | rs29481846 | Celera2   mCV22906380 |
| 11 5.795469 |  |  | *Myl7* L | C | A | rs29414291 | Celera2   mCV22906390 |
| 11 5.798794 |  |  | *Myl7* L | C | G | rs26914598 | (Celera2,Perlegen2) |
|  |  |  |  |  |  |  |  |
| ***Npc1*** |  |  |  |  |  |  |  |
| 18 12.346453 | [3110002H16Rik   intron14](javascript:;) | agrees | *Npc1* L | G | A | rs30994669 | (CGD2,Perlegen2) |
|  |  |  | 3110002H16Rik I |  |  |  |  |
| 18 12.347229 | [3110002H16Rik   intron16](javascript:;) | agrees | 3110002H16Rik I | G | A | rs30993687 | Perlegen2   NES12708472 |
|  |  |  | *Npc1* L |  |  |  |  |
| 18 12.347455 | [3110002H16Rik   intron17](javascript:;) | agrees | *Npc1* L | C | T | rs29878990 | (Broad1,Broad2,CGD2,Chicago1,Perlegen2) |
|  |  |  | 3110002H16Rik I |  |  |  |  |
| 18 12.348019 |  |  | 3110002H16Rik I | C | A | rs30993684 | Perlegen2   NES12708475 |
|  |  |  | *Npc1* L |  |  |  |  |
| 18 12.349168 | [*Npc1*   UTR](javascript:;) | ... exon25,UTR | U | A | G | rs30992818 | Perlegen2   NES12708350 |
| 18 12.349329 | [*Npc1*   UTR](javascript:;) | ... exon25,UTR | U | T | G | rs30992814 | Perlegen2   NES12708357 |
| 18 12.350153 | [*Npc1*   intron24](javascript:;) |  | I | T | C | rs30991093 | Perlegen2   NES12708268 |
| 18 12.351367 | [*Npc1*   intron23](javascript:;) | agrees | I | A | G | rs30991089 | (CGD2,Perlegen2) |
| 18 12.351403 | [*Npc1*   intron23](javascript:;) | agrees | I | G | C | rs30991088 | (CGD2,Perlegen2) |
| 18 12.351430 | [*Npc1*   intron23](javascript:;) | agrees | I | A | G | rs30991086 | Perlegen2   NES12708137 |
| 18 12.351540 | [*Npc1*   intron23](javascript:;) | agrees | I | T | C | rs30990270 | Perlegen2   NES12708142 |
| 18 12.351914 | [*Npc1*   exon23](javascript:;) | agrees | **Cs**   S1158 | A | G | rs30990267 | (CGD2,Perlegen2) |
| 18 12.351932 | [*Npc1*   intron22](javascript:;) | agrees | I | A | C | rs30990265 | Perlegen2   NES12708145 |
| 18 12.352443 | [*Npc1*   intron22](javascript:;) | agrees | I | T | C | rs30989389 | (CGD2,Perlegen2) |
| 18 12.352596 | [*Npc1*   exon22](javascript:;) | agrees | **Cs**   A1149 | A | C | rs30989385 | (CGD2,Perlegen2) |
| 18 12.353082 | [*Npc1*   intron21](javascript:;) | agrees | I | T | C | rs30988587 | Perlegen2   NES12708032 |
| 18 12.353393 | [*Npc1*   intron21](javascript:;) | agrees | I | C | T | rs30988087 | (CGD2,Perlegen2) |
| 18 12.354104 | [*Npc1*   intron20](javascript:;) | agrees | I | G | T | rs30989455 | (CGD2,Perlegen2) |
| 18 12.354471 | [*Npc1*   intron20](javascript:;) | agrees | I | C | T | rs30988681 | Perlegen2   NES12707904 |
|  |  |  |  |  |  |  |  |
| ***Npc1l1*** |  |  |  |  |  |  |  |
| 11 6.121546 | [*Npc1l1*   intron10](javascript:;) | agrees | I | G | A | rs29445501 | Celera2   mCV24401705 |
| 11 6.126402 | [*Npc1l1*   intron2](javascript:;) | agrees | I | A | G | rs29400944 | Celera2   mCV24401706 |
| 11 6.127984 | [*Npc1l1*   exon2](javascript:;) | agrees | **Cn**   K475N | G | T | rs29453506 | Celera2   mCV24401707 |
| 11 6.128736 | [*Npc1l1*   exon2](javascript:;) | agrees | **Cn**   G225C | A | C | rs26899815 | (Celera2,Perlegen2) |
| 11 6.128977 | [*Npc1l1*   exon2](javascript:;) | agrees | LOC100041242 L | T | C | rs26899814 | (Broad1,Broad2,CGD2,Celera2,Perlegen2) |
|  |  |  | *Npc1l1* **Cs**   R144 |  |  |  |  |
| 11 6.129461 | [*Npc1l1*   intron1](javascript:;) | agrees | LOC100041242 L | G | C | rs26899813 | (Celera2,Perlegen2) |
|  |  |  | *Npc1l1* I |  |  |  |  |
